# Integration of Bulk and Single-Cell RNA-Seq Data to Construct a Prognostic Model of Membrane Tension-Related Genes for Colon Cancer

**DOI:** 10.3390/vaccines10091562

**Published:** 2022-09-19

**Authors:** Jiacheng Li, Yugang Fu, Kehui Zhang, Yong Li

**Affiliations:** 1 Shanghai Municipal Hospital of Traditional Chinese Medicine, Shanghai University of Traditional Chinese Medicine, Shanghai 200071, China; 2 Municipal Medical College of Traditional Chinese Medicine, Shanghai University of Traditional Chinese Medicine, Shanghai 200071, China

**Keywords:** membrane tension, colorectal cancer, prognostic signature, immune microenvironment, molecular docking

## Abstract

Background: The plasma membrane provides a highly dynamic barrier for cancer cells to interact with their surrounding microenvironment. Membrane tension, a pivotal physical property of the plasma membrane, has attracted widespread attention since it plays a role in the progression of various cancers. This study aimed to identify a prognostic signature in colon cancer from membrane tension-related genes (MTRGs) and explore its implications for the disease. Methods: Bulk RNA-seq data were obtained from The Cancer Genome Atlas (TCGA) database, and then applied to the differentially expressed gene analysis. By implementing a univariate Cox regression and a LASSO-Cox regression, we developed a prognostic model based on four MTRGs. The prognostic efficacy of this model was evaluated in combination with a Kaplan–Meier analysis and receiver operating characteristic (ROC) curve analysis. Moreover, the relationships between the signature and immune cell infiltration, immune status, and somatic mutation were further explored. Lastly, by utilizing single-cell RNA-seq data, cell type annotation, pseudo-time analysis, drug sensitivity, and molecular docking were implemented. Results: We constructed a 4-MTRG signature. The risk score derived from the model was further validated as an independent variable for survival prediction. Two risk groups were divided based on the risk score calculated by the 4-MTRG signature. In addition, we observed a significant difference in immune cell infiltration, such as subsets of CD4 T cells and macrophages, between the high- and low-risk groups. Moreover, in the pseudo-time analysis, TIMP1 was found to be more highly expressed with the progression of time. Finally, three small molecule drugs, elesclomol, shikonin, and bryostatin-1, exhibited a binding potential to TIMP-1. Conclusions: The novel 4-MTRG signature is a promising biomarker in predicting clinical outcomes for colon cancer patients, and TIMP1, a member of the signature, may be a sensitive regulator of the progression of colon cancer.

## 1. Introduction

Colon cancer remains one of the world’s most common intestinal diseases; it ranks third in terms of incidence, but second in terms of mortality worldwide [1]. In 2020, an estimated 104,610 new cases of colon cancer and 43,340 cases of rectal cancer were predicted to have occurred in the United States [2]. Due to multiple factors, such as diet, lifestyle, and a lack of health-care infrastructure and resources, the incidence and mortality of colon cancer remain on the rise. We are in an era of the rapid development of cancer-screening methods; however, many patients are diagnosed at an advanced stage and suffer a poor survival rate with only a few effective therapeutic targets for colon cancer [3]. Immune checkpoint inhibitor (ICI) drugs and CAR-T cell therapy have provided durable clinical benefits for metastatic colon cancer [4,5]. Therefore, apart from improvements in chemoradiotherapies and surgical instruments, it is also crucial to develop novel diagnostic and prognostic models, as well as possible therapeutic targets, from emerging sequencing data and computational tools.

Membrane tension, defined as the force per unit length acting on a cross-section of the membrane, regulates many vital biological processes [6]. It consists of two parts: one from the in-plane tension in the lipid bilayer, the other from the adhesion between the membrane and the cytoskeleton [7]. Membrane tension organizes a cell’s shape and its motility, regulating the balance between exocytosis and endocytosis [8]. It is known that derailed endocytosis can majorly contribute to several hallmarks of cancer, not only the sustained proliferation of cancer cells but also its enhanced invasiveness and avoidance of apoptosis [9]. A recent study found that reduced membrane tension is a mechanical hallmark of malignant cells. Cells with lower membrane tension levels are more susceptible to tumor invasion and metastasis [10]. Moreover, researchers found that membrane tension could regulate glycolytic rates in cancer cells, shedding light on how a tumor transforms from stiff to soft [11]. However, MTRGs in colon cancer have not yet been identified. Thus, clinical sample-based screenings for MTRGs are necessary for their possible value in the diagnosis and treatment of colon cancer.

## 2. Materials and Methods

### 2.1. Data Acquisition

The bulk RNA-seq data were obtained from the TCGA database (https://tcga-data.nci.nih.gov/tcga/, accessed on 18 April 2022) by a thorough examination of COAD-related datasets. Samples from 473 COAD patients were used for further analysis. All samples were normalized by the fragments per kilobase million (FPKM). A total of 427 patients in the cohort provided information on gene mutations and corresponding clinical characteristics, including age, gender, TNM stage, and survival status, which were also extracted from TCGA. A detailed description of the clinical characteristics of these patients is shown in Supplementary Table S1. In addition, the GSE14333 and GSE103479 cohorts were also obtained from the Gene Expression Omnibus (GEO) (https://www.ncbi.nlm.nih.gov/, accessed on 18 April 2022) database as independent external cohorts for prognostic model validation. The scRNA-seq data were downloaded from GSE161277, including three normal samples (N1, N2, and N3) and three tumor samples (T1, T2, and T3). The tumor samples included 4118, 4383, and 4137 cells, and the normal samples included 3868, 7549, and 4184 cells. A total of 44 MTRGs were retrieved from previously published literature [10,12,13,14,15,16,17,18,19], whose details have been provided in Supplementary Table S2.

### 2.2. Construction and Validation of the MTRG Prognostic Model

**Figure 1:**
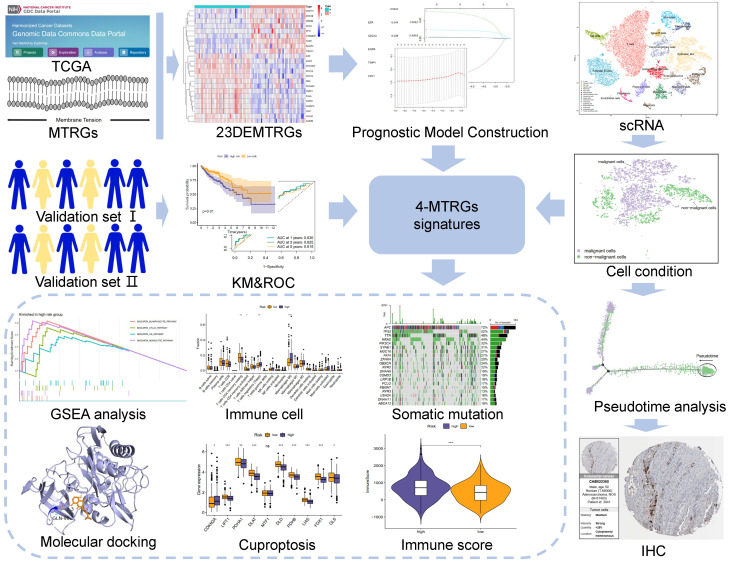
Flowchart of this study. * *p* < 0.05, ** *p* < 0.01, *** *p* < 0.001.

The prognostic risk of each differentially expressed MTRG was firstly assessed using univariate Cox regression, and then features with *p*-values < 0.05 in the training cohort were defined as prognostic factors. Next, the optimal gene combinations were screened out by least absolute shrinkage and selection operator (LASSO) regression and multivariate Cox analysis to construct the risk score model. The risk score was calculated as the sum of the products of gene expression levels and their coefficients. Subjects were divided into high- and low-risk groups based on the median risk score as a threshold. Then, Kaplan–Meier analysis and ROC curve analysis were applied to assess the prognostic role of the model. We performed univariate and multivariate Cox regression analyses to determine whether risk scores could be an independent prognostic factor for COAD patients using the “survival” R package. As mentioned above, two independent cohorts from the GSE14333 and GSE103479 were used to verify the utility of the prognostic model (Figure 1).

### 2.3. Visualization of Mutation and CNV Data

Two waterfall diagrams were depicted with the R package “maftools” [20] to explore the somatic mutations between the high- and low-risk groups of COAD patients. Kaplan–Meier analysis was also performed to compare the survival risks between the two groups. Copy Number Variation (CNV) data of the model genes were downloaded from USCS Xena (https://xenabrowser.net/datapages/, accessed on 18 April 2022). Genes in the CNV region were annotated by the Genome Research Consortium Human build 38 (GRCh38) as the reference genome.

### 2.4. Protein Interaction Network Construction and GSEA Analysis

GeneMANIA (http://genemania.org/, accessed on 18 April 2022) is an online tool for predicting the interactions and functions of genes and gene sets [21]. In the present study, a 4-MTRG-involved protein interaction network was constructed using this web tool, and other potential proteins associated with MTRG were screened and predicted. Then, the “org.Hs.eg.db”, “clusterProfiler”, and “enrichplot” R packages were used to perform Gene Set Enrichment Analysis (GSEA) to identify biologically significant enrichment pathways between high- and low-risk groups.

### 2.5. Immune Cell Infiltration, Gene Set Enrichment Analysis, and ICI-Related Gene Expression between the High- and Low-Risk Groups

To clarify the immune status between different groups, 22 types of immune cells were identified in each sample by the CIBERSORT algorithm [22]. The infiltration density of these immune cells was calculated groupwise with the R package “limma”. Then, a single-sample gene set enrichment analysis (ssGSEA) was performed using the “GSVA” and “GSEABase” R packages to assess the infiltration fraction of the known 13 immune cell genomes and the known 16 immune activities of immune-related pathways, respectively. The results were calibrated to the range between 0 and 1. The correlation between tumor microenvironment and risk scores was assessed by the stromal score, ESTIMATE score, immune score, and tumor purity obtained from previous ESTIMATE calculations for each sample. In addition, we analyzed the expression levels of immune checkpoint inhibitors (ICIs) and cuproptosis genes between the two groups.

### 2.6. Dimensionality Reduction, Clustering, and Annotation of scRNA-Seq Data

“Seurat” R package was adopted to convert previously downloaded 10× scRNA-seq data as a Seurat object. All functions below are inherited from Seurat. Quality control was performed on the raw counts by calculating the percentage of mitochondrial and erythrocyte genes and by excluding low-quality cells, followed by homogenization using the “NormalizeData function”. Top 3000 highly variable features were filtered using the “FindVariableFeatures” function, and normalization was completed by the “ScaleData” function. Principal component analysis (PCA), a preliminary linear dimensionality reduction method, was performed on the scaled data with the elimination of the batch effect using Harmony (v1.0) by default. The t-SNE algorithm, a nonlinear dimensionality reduction technique, was performed for cluster identification. Biologically significant cell types were annotated by the “FindAllMarkers” function to find representative genes for each cluster in combination with typical cell markers.

### 2.7. Subclusters of Major Cell Types and Pseudo-Time Analysis

To validate the expression of the genes at a single cell transcriptome level, we re-clustered the epithelial cell subgroups with the same method. To determine the lineage of non-malignant cells and malignant cells, the “infercnv” R package was used to determine the malignant clusters. The “monocle” R package was adopted for cell trajectory and pseudo-time analysis, with the method “DDRTree” used for dimension reduction. A heatmap was made to visualize the expression level of MTRGs, and a scatter plot was plotted at last to visualize the change in relative gene expression of the 4-MTRG signature over time.

### 2.8. Validation of Protein Expression by the HPA Database

Immunohistochemistry (IHC) images from the Human Protein Atlas database (HPA, https://www.proteinatlas.org/, accessed on 18 April 2022) were adopted to validate the protein expression of the MTRGs from the prognostic model. The expression level of TIMP1 was compared between tumor tissues and normal tissues.

### 2.9. Molecular Docking

The “pRRophetic” R package was used to analyze the differences in drug sensitivity between the high- and low-risk groups. It was further used to screen for relevant active ingredients. The structural formulae of the active ingredients were downloaded from the PubChem (https://pubchem.ncbi.nlm.nih.gov/, accessed on 18 April 2022) database. Chem3D software was used to create 3D structures of the active ingredients. The 3D structures of MTRGs were downloaded from the PDB database (http://www.rcsb.org/, accessed on 18 April 2022). PyMOL software (https://pymol.org/2/, accessed on 18 April 2022) was used to perform operations such as dehydration and hydrogenation of proteins. In addition, AutoDockTools (v1.5.7) software was used to search for ligand-binding pockets. Subsequently, the Vina script was applied to calculate the molecular binding energy and display the molecular docking results. Finally, the results were imported into PyMOL software for visualization.

### 2.10. Statistical Analysis

Wilcoxon sign tests were used to compare the relationship between two groups for continuous variables. Cox and LASSO-Cox regression were used for predictable models. Kaplan–Meier analyses were used to test survival differences between different risk groups. The Spearman test was used to compare qualitative variables if the value of the variable was small. A two-sided *p*-value < 0.05 was considered significant. All statistical analyses were performed using R software (version 4.1.3).

## 3. Results

### 3.1. Identification of 23 Differentially Expressed MTRGs

**Figure 2:**
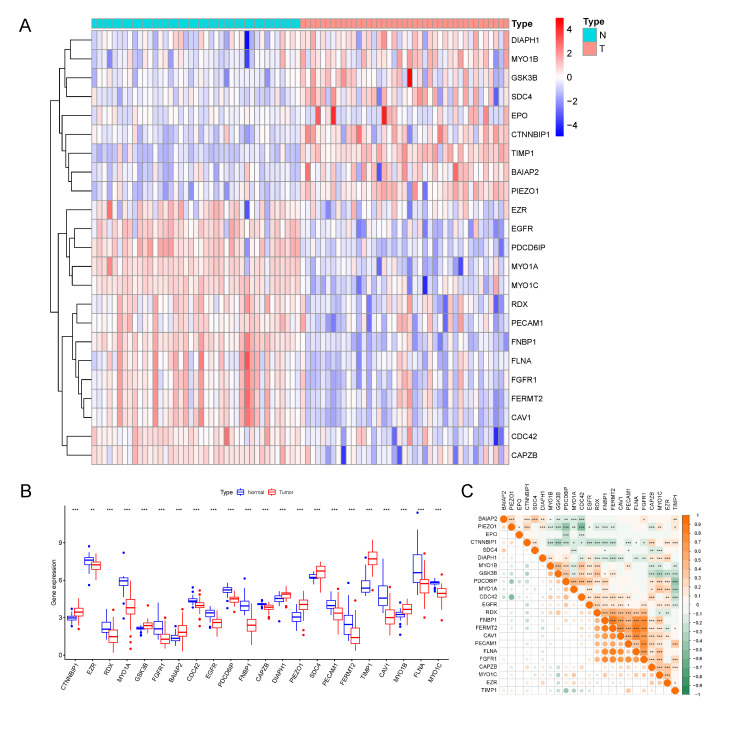
Differentially expressed MTRGs in COAD tissues compared with normal colon tissues and their interactions. (**A**) Heatmap of the expression of the 23 MTRGs in the tumors and normal tissues of the TCGA dataset. (**B**) The expression of MTRGs was significantly different in 41 COAD compared with the normal colon pairs. (**C**) Interaction analysis among the 23 MTRGs. * *p* < 0.05, ** *p* < 0.01, *** *p* < 0.001.

To determine the expressional changes of MTRGs in the COAD patients, we quantified the MTRG expression from previously downloaded bulk RNA-seq data from TGCA. We used 41 normal samples and randomly chose 41 out of 473 samples from the COAD patients. The differential analysis reported that the expression levels of MTRGs between the tumor and normal samples were distinct (Figure 2A). A total of 23 MTRGs (9 up and 14 down) were finally identified as differentially expressed genes in the COAD group compared with the normal group (Figure 2B; *p* < 0.05). Additionally, we examined the correlation among the 23 MTRGs, where FLNA, FNBP1, FGFR1, FERMT2, and CAV1 showed a strong correlation between each other (cutoff > 0.85) (Figure 2C).

### 3.2. Construction and Validation of a 4-MTRG Prognostic Model

**Figure 3:**
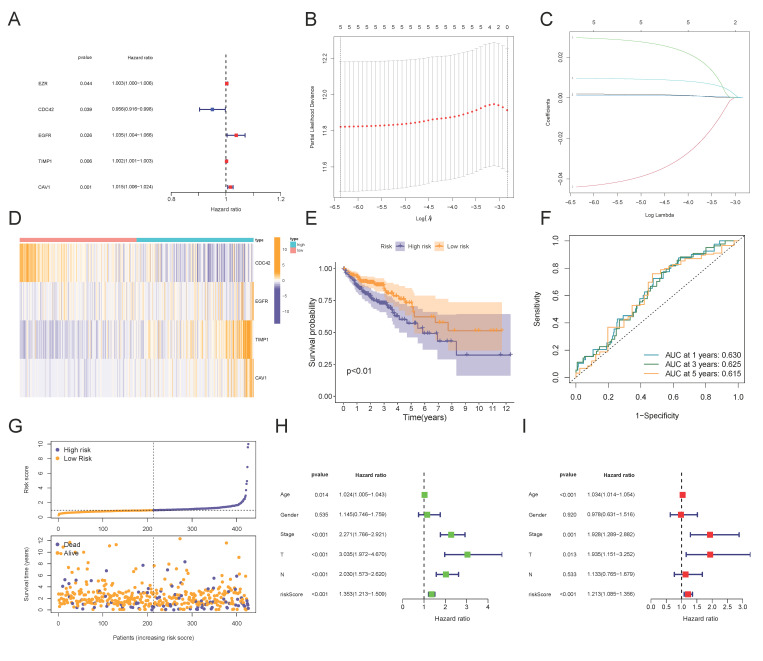
Construction of the prognostic risk model based on the TCGA-COAD cohort: (**A**) Forest map of 5 MTRGs significantly correlated with OS, identified by univariate cox analysis. (**B**) Screening of optimal parameters (lambda) in the LASSO regression model based on the TCGA cohort. (**C**) LASSO coefficient profiles of the 4 MTRGs determined by the optimal lambda. (**D**) Heatmap of the expression of 4 MTRGs in high- and low-risk groups. (**E**) Kaplan–Meier curve for the OS of colon cancer patients in the high- and low-risk groups in the TCGA cohort. (**F**) ROC analysis of the prognostic model for OS and survival status in the TCGA cohort. (**G**) Scatterplots in the top and bottom illustrate the distribution of the risk score and survival status in the colon cancer patients, respectively. (**H**,**I**) Univariate (**H**) and multivariate (**I**) Cox regression analyses of the risk score and clinicopathological parameters in the TCGA cohort.

First, we applied a univariate Cox regression to evaluate the prognostic effects of the 23 differentially expressed MTRGs; thus, five genes (EZR, CDC42, EGFR, TIMP1, CAV1) were retained (Figure 3A). Then, LASSO–Cox regression analysis was applied to five candidate genes (Figure 3B,C). Ultimately, we constructed an optimal prognostic model with a 4-MTRG signature consisting of CDC42, EGFR, TIMP1, and CAV1 (Supplementary Table S3). A total of 427 COAD patients were assigned to our prognostic model. Then, we calculated the risk score (RS) of each patient according to the following formula:

(1) RS=(−0.043)×CDC42+0.032×EFGR+0.001×TIMP1+0.011×CAV1

**Figure 4:**
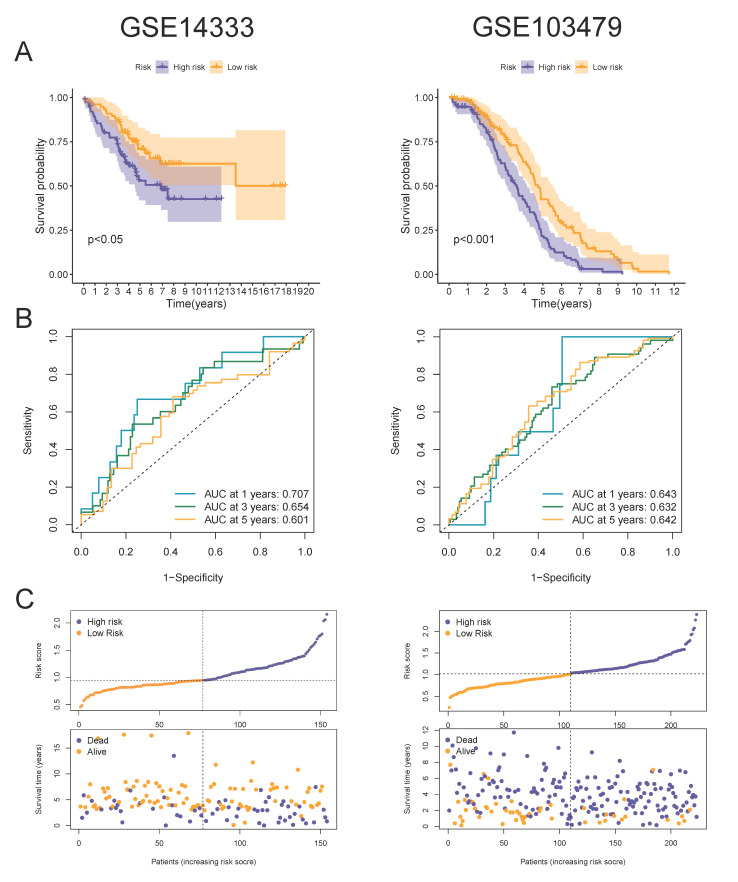
External validation of prognostic risk models: (**A**) Kaplan–Meier survival analysis of OS between patients with high-risk scores and low-risk scores in the GSE14333 and GSE103479 cohorts. (**B**) ROC analysis of the prognostic model in the GSE14333 and GSE103479. (**C**) risk score and survival status in the GSE14333 and GSE103479 cohorts.

Next, all patients were divided into high- and low-risk groups according to the median value of the risk scores. We made a heatmap of the expression level of the 4-MTRG signature in the high- and low-risk groups. The expression of EGFR, TIMP1, and CAV1 was higher in the high-risk group than in the low-risk group, while the expression of CDC42 was the opposite (Figure 3D). The Kaplan–Meier analysis showed that patients in the high-risk group were associated with a worse overall survival (OS) if compared with patients in the low-risk group (Figure 3E). The receiver operating characteristic (ROC) curve showed the potential of the prognostic model in predicting 1-, 3-, and 5-year OS in the entire cohort (areas under the curve are 0.630, 0.625, and 0.615 (Figure 3F)). We also depicted the distribution of the risk score and survival status among the patients (Figure 3G). Moreover, univariate and multivariable Cox regression analyses were utilized to identify whether the model-derived risk score could be an independent predictor of OS. The results of the univariable regression showed that age, AJCC stage, T stage, N stage, and risk score were closely related to OS (Figure 3H; *p* < 0.05). Similarly, in the multivariable regression, age, AJCC stage, T stage, and risk score maintained their prognostic power (Figure 3I; *p* < 0.05). In brief, these data demonstrated that the risk score serves as an independent indicator to predict prognoses for patients in the TGCA cohort. Subsequently, we observed similar results in two external cohorts, GSE14333 and GSE103479 (Figure 4A–C). The Kaplan–Meier analysis and ROC curves of each cohort both suggested that the patients from the high-risk group present a lower OS compared with the low-risk group, which validated the robustness and validity of the prognostic model.

### 3.3. Somatic Mutation Profile of the Tumor Samples

**Figure 5:**
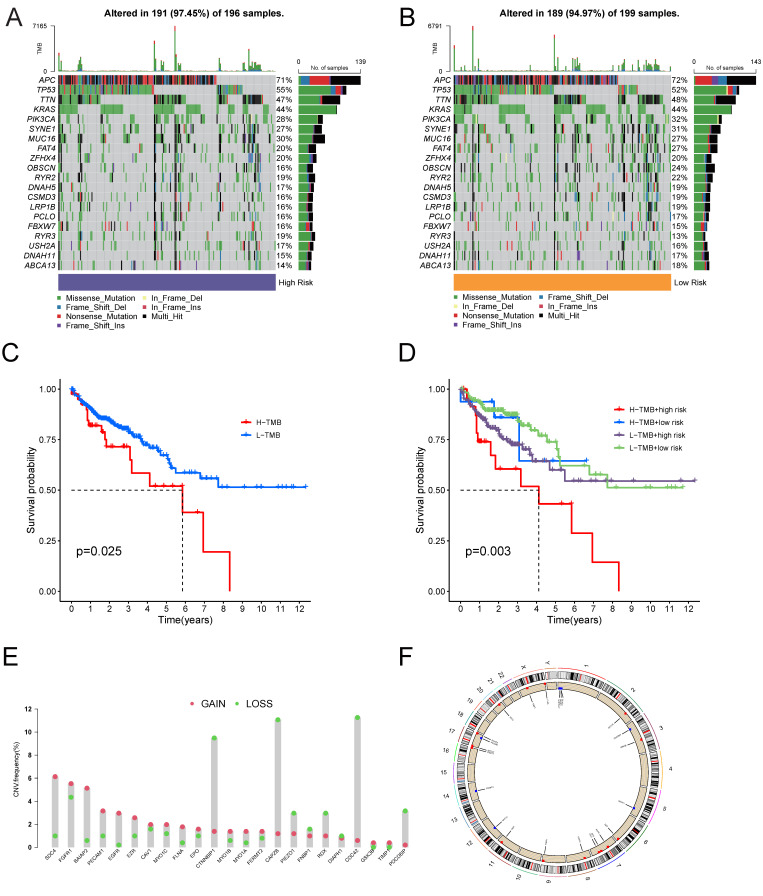
Somatic mutation and CNV analysis in colon cancer patients: (**A**,**B**) Waterfall plots illustrate the gene mutation landscape in high- and low-risk groups. (**A**) High-risk group; **(B**) low-risk group. (**C**) Survival analysis of OS in colon cancer patients between high- and low-TMB groups. (**D**) Survival analysis of OS in colon cancer patients between high- and low-TMB groups based on risk score. (**E**) CNV frequency of 23 MTRGs. (**F**) Genomic position of 23 MTRGs. Bands at the inner circle indicate corresponding expression levels.

Previously, we divided patients into two groups based on the median risk score calculated by the 4-MTRG prognostic model; however, we have not obtained the genomic profiling of the tumor samples regarding the somatic alterations that drive cancer progression and patient survival. Here, the copy number variation (CNV) profile is represented group-wise using waterfall graphs. (Figure 5A,B). Both groups showed high mutation alteration rates (97.45% for the high-risk group and 94.97% for the low-risk group). Moreover, we reassigned the patients into two groups based on the median of the tumor mutation burden (TMB), an index that indicates the level of somatic mutation in tumor cells. The high TMB group showed a poorer OS than the low TMB group in the Kaplan–Meier analysis (Figure 5C). When the TMB and prognostic risk score were both included in the survival analysis model, the combination of a high TMB and a high risk behaved the worst (Figure 5D). Next, we concentrated on the frequency of the CNVs of the MTRGs. The CNV analysis of the 23 MTRGs suggested that part of the cleavage enzymes had frequent copy number deletions (Figure 5E), and an overall visualization of the genomic position of the MTRG-related CNV is provided in Figure 5F.

### 3.4. Protein Interaction Network of the 4-MTRG Signature and GSEA Analysis

**Figure 6:**
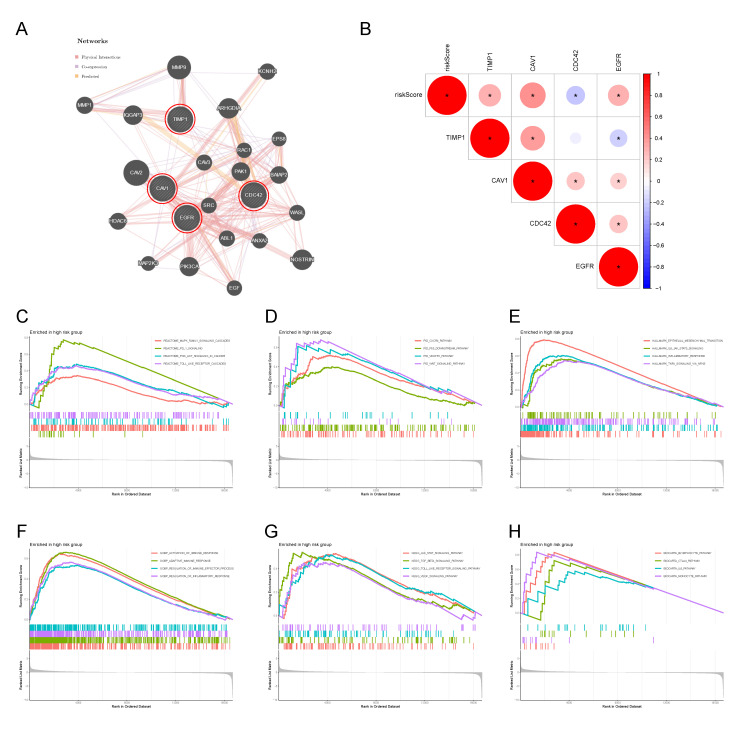
Network regulation and functional enrichment analysis of high-risk groups based on MTRGs prognostic signature. (**A**) The regulatory network involving 4-MTRGs and twenty potential binding proteins was constructed through GeneMANIA. (**B**) Correlation analysis of 4 genes. * *p* < 0.05 (**C**–**H**) GSEA showed the significantly enriched Reactome (**C**), PID (**D**), Hallmark (**E**), GOBP **(F**), KEGG (**G**), and BioCarta (**H**) gene sets in the high-risk colon cancer patients.

Here we constructed a protein interaction network for the 4-MTRG signature from the prognostic model (Figure 6A). The regulatory network carried 24 genes, including four MTRGs and an additional twenty genes that have the potential to interact with them. The additional genes were predicted and added automatically through “GeneMANIA”. The correlation between the risk score and the 4-MTRG signature is shown in Figure 6B. Furthermore, we applied GSEA analysis to investigate the relevant biological processes and signaling pathways in the high-risk group (Figure 6C–H). The results show that cancer hallmark-based gene sets, such as epithelial–mesenchymal transition signaling pathway and the JAK–STAT3 signaling pathway, were highly enriched in the high-risk group. Moreover, the high-risk group is involved in biological processes such as the activation of the immune response, adaptive immune response, and inflammatory response. Furthermore, several classical pathways from the KEGG, Reactome, BioCarta, and PID gene sets, including the toll-like receptor pathway, monocyte pathway, TGF-β pathway, PD-1-signaling pathway, and PI3K-AKT pathway, were also highly related to the high-risk group.

### 3.5. Risk Scores Related to Different Immune Cell Infiltration, Immune Status, and ICIs

**Figure 7:**
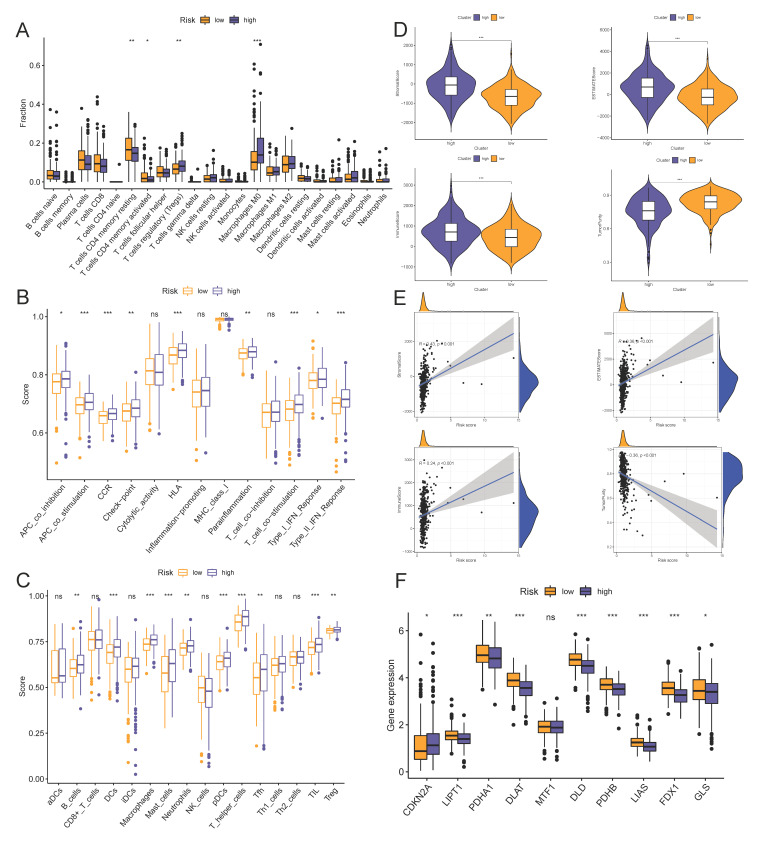
The high- and low-risk groups present different immune statuses. (**A**) The boxplots for the comparison of the 22 immune cells between the high-risk and low-risk groups in colon cancer. (**B**,**C**) Immune cells infiltration score (**B**) and immune-related pathways’ activity (**C**) in the high- and low-risk groups estimated by ssGSEA. (**D**) Expression level of the stromal score, ESTIMATE score, immune score, and tumor purity in the high- and low-risk groups. (**E**) Associations between the risk score and immune cell infiltration levels. (**F**) Cuproptosis-related genes between the high- and low-risk groups. * *p* < 0.05, ** *p* < 0.01, *** *p* < 0.001.

The interaction between the risk genes and the immune status was of a certain prognostic significance for colon cancer. We depicted the immune cell infiltration landscape of the two groups with the CIBERSOFT algorithm. Then, we compared the infiltration density of the immune cells between the high- and low-risk groups. The results suggested that activated CD4^+^ memory T cells and resting CD4^+^ memory T cells were downregulated in the high-risk group, while regulatory T cells and M0 macrophages were upregulated in the high-risk group (Figure 7A). Moreover, ssGSEA was performed to evaluate immune activity towards 13 immune cell types and 16 relative immune pathways, and the enrichment analysis output was further compared between the two groups. We found that immune cells in the high-risk group had a more active behavior, in which the numbers of B cells, dendritic cells, macrophages, mast cells, neutrophils, plasmacytoid dendritic cells, T helper cells, follicular helper T cells, tumor-infiltrating lymphocytes, and regulatory T cells were significantly higher than the low-risk group (Figure 7B). Moreover, 7 of 13 immune pathways reported significant differences, especially the APC co-stimulation pathway, CCR pathway, HLA pathway, T cell co-stimulation pathway, and type II IFN response pathway (*p* < 0.001; Figure 7C). To further validate this finding, we applied four tumor microenvironment-related scoring approaches to evaluate the differences of immune status between the two groups. In total, four scores, including the stromal score (substrate cells in the tumor tissue), immune score (immune cell infiltration in the tumor tissue), and estimate score (the summation of stromal and immune scores from individual cases) were adopted. We found that the estimate score, immune score, and stromal score were all significantly higher in the high-risk group, whereas the tumor purity scores were lower in the high-risk group. (*p* < 0.001; Figure 7D). We also provided a scatter plot to elucidate the correlation between the risk score and the four scores. The estimate score, immune score, and stromal score showed a positive correlation with the risk score, while the tumor purity score showed a negative correlation (Figure 7E). Recently, by taking advantage of cutting-edge tumor immunology, scientists have developed novel drugs targeted at ICIs for treating solid tumors, and a few products targeted at CTLA4 and PD-1 have been put into clinical practice. To further understand the relationship between our prognostic model and ICIs, 25 ICIs were analyzed between the two groups. We found that risk scores had a positive correlation with the expression of ICIs, and all the expression levels of ICIs showed a statistical significance between groups (Supplementary Figure S1). Cuproptosis, a novel, regulated cell death distinct from known mechanisms broadens our knowledge of the homeostatic conditions of the cell [23], and may be involved in the progression of gastrointestinal tumors; hence, we analyzed the expression level of cuproptosis-related genes between the groups. Ten genes were analyzed, eight were upregulated and one downregulated in the high-risk group (Figure 7F), indicating that the cell might have been in a highly active mode and the death mechanism might have been inhibited.

### 3.6. scRNA-Seq and Cell Type Characteristics of Normal and COAD Samples

**Figure 8:**
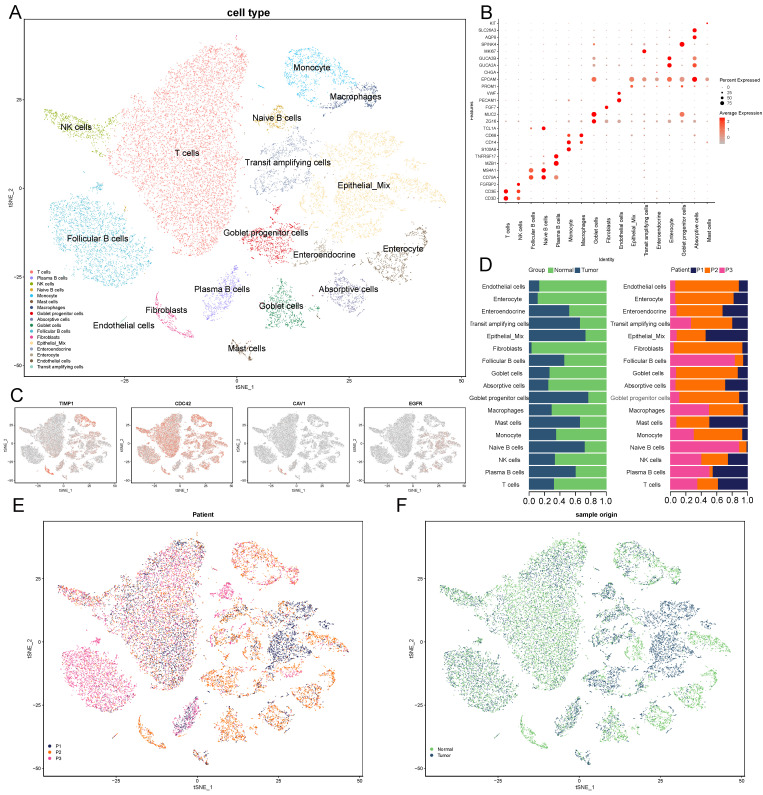
Cell type constitution of the colon normal and adenoma tissues. (**A**) t-SNE plots of cells from three patients (6 samples). Colors represent cell types. Cells were clustered into 17 sub-clusters based on biological annotation. Each dot represents a single cell. (**B**) Dot plot of proportion of cells in the respective cluster expressing selected marker genes. Circle size represents the percentage of cells that express the gene, and color represents the average expression value within a cluster. (**C**) Expression of the 4-MTRGs at the single cell level. (**D**) Bar plot showing the fraction of cells originating from the 3 normal and 3 tumor samples (left) and the fraction of cells originating from each of the 3 patients (right). (**E**,**F**) t-SNE plots of patient origin (**E**) and sample origin (**F**).

We downloaded 10× scRNA-seq data of three tumor samples and three normal samples from the GSE161277 dataset. A total of 23,634 cells were identified after quality control. The top 3000 highly variable characteristics were selected after normalization; the batch effect was calibrated with the “Harmony” package. We then identified the typical markers of 25 distinct clusters after a PCA and t-SNE analysis, and 17 clusters were shown after merging and annotating manually (Figure 8A). Then, we annotated the clusters based on several canonical marker genes for known cell lineages: T cell (CD3D), NK cell (FGFBP2), follicular B cell (MS4A1), native B cell (TCL1A), plasma B cell (MZB1), monocyte (CD14), macrophage (CD68), goblet cell (MUC2), fibroblast (DCN), endothelial cell (VWF), epithelial mix (EPCAM and PROM1), transit-amplifying cell (MKI67), enteroendocrine (CHGA), enterocyte (GUCA2A and GUCA2B), goblet-progenitor cell (SPINK4), absorptive cell (AQP8 and SLC26A3), and mast cell (KIT) (Figure 8B). To obtain a general view of the distribution of the four MTRGs from the prognostic model, we mapped their distribution with a t-SNE plot. The result showed that CDC42, a regulator of both the architecture and motility of the plasma membrane, appeared most broadly; TIMP1 mostly appeared in epithelial mix, macrophages, and monocytes; and CAV1 and EGFR were distributed sparsely (Figure 8C). Notably, epithelial cells were the only cell type that all the 4-MTRGs exhibited expression in. Next, we labeled the epithelial mix either by patient number (P1–P3) or sample type (normal or tumor). Then, we made bar charts (Figure 8D) to present the proportion of the 17 cell types categorized by their labels. We also made t-SNE plots to present the proportion that each label accounts for in the whole area. We could see that a large portion of epithelial mix, transit-amplifying cells, and goblet progenitor cells were from the tumor samples, and the distribution of the different cell types among the patients were heterogeneous (Figure 8E,F).

### 3.7. Subclusters of the Epithelial Cell and Pseudo-Time analysis

**Figure 9:**
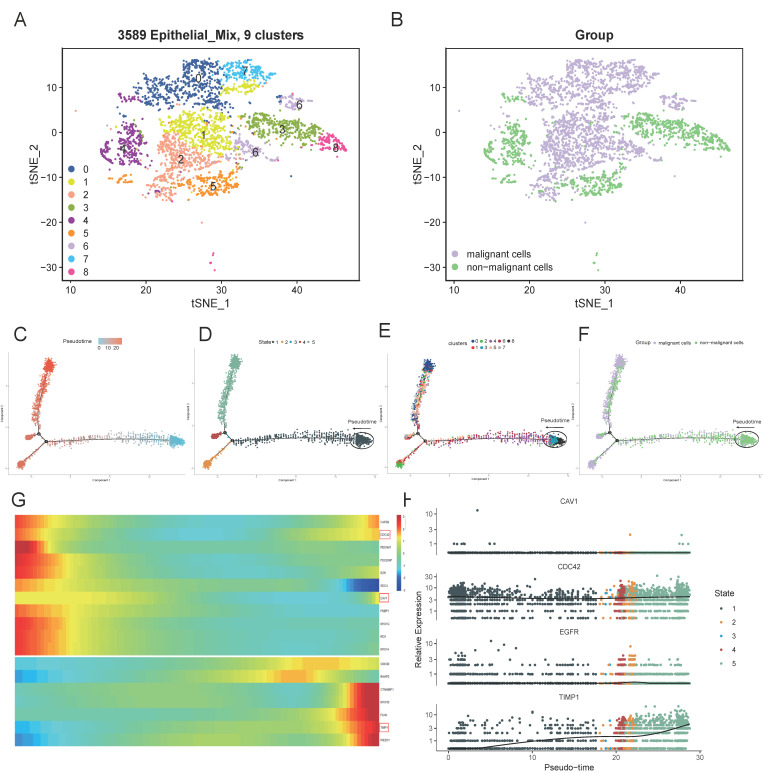
Epithelial cell clusters’ and pseudotime analysis. (**A**,**B**) t-SNE plot of 3589 epithelial cells, color-coded by their associated cluster (**A**) or cell classification (**B**). Note that the cell type was judged to be malignant by inferCNV analysis (Supplementary Figure S2). (**C**–**F**) Trajectory reconstruction (**C**) of all single cells from non-malignant to malignant epithelial cells; states (**D**), distribution of clusters (**E**), and cell types (**F**) on the trajectory are shown. (**G**) Heatmap showing time-series gene expression (rows) in epithelial cells (columns) for each membrane tension-related gene. (**H**) Gene expression dynamics of 4-MTRGs are displayed.

**Figure 10:**
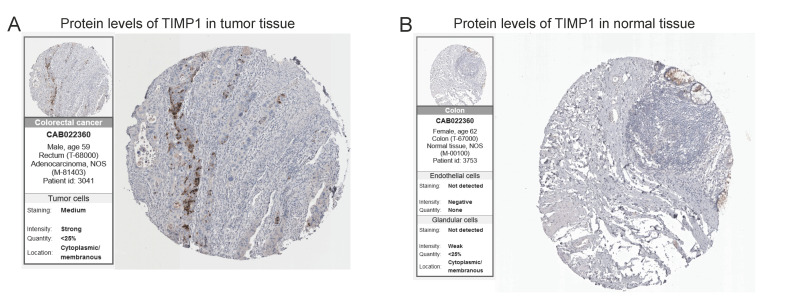
Immunohistochemistry of the TIMP1 gene in tumor and normal tissues from the human protein atlas (HPA) database. (**A**) Protein levels of TIMP1 in tumor tissue. (**B**) Protein levels of TIMP1 in normal tissue.

As we know from the descriptive analysis of the previous section, all the four prognostic model-derived MTRGs expressed in the epithelial mix. Therefore, we re-clustered the epithelial mix following the same procedure; the mix was divided into nine clusters (Figure 9A). Then, the “infercnv” R package was used to estimate the proportion of malignant cells based on the clusters. A total of 62.8% of the cells of the epithelial mix were predicted to be malignant (Figure 9B; Supplementary Figure S2). Then, the “monocle” R package was exploited to analyze the cell trajectory and pseudo-time of the epithelial mix. We observed that the epithelial mix was present in all stages and transformed into malignant cells along the timeline (Figure 9C–F). In addition, we created a heatmap to visualize the expression levels of all 23 MTRGs in the epithelial cells; 18 genes reported expressional changes with statistical significance (Figure 9G). Then, we tested the relative expressions of the 4-MTRG signature in the epithelial mix and found that only the expression of TIMP1 had a higher relative increase than at initiation (Figure 9H). With the help of the HPA database, we identified that TIMP1 has a high expression in colorectal tumor tissue (Figure 10). Overall, we found that three genes in the prognostic model (CDC42, CAV1, and TIMP1) had an increasing degree of expression in the epithelial mix from the patients’ samples over time, among which the expression of TIMP1 had a threefold increase at the final stage.

### 3.8. Drug Sensitivity of the Two Groups and Docking of Drug Candidates to TIMP1

**Figure 11:**
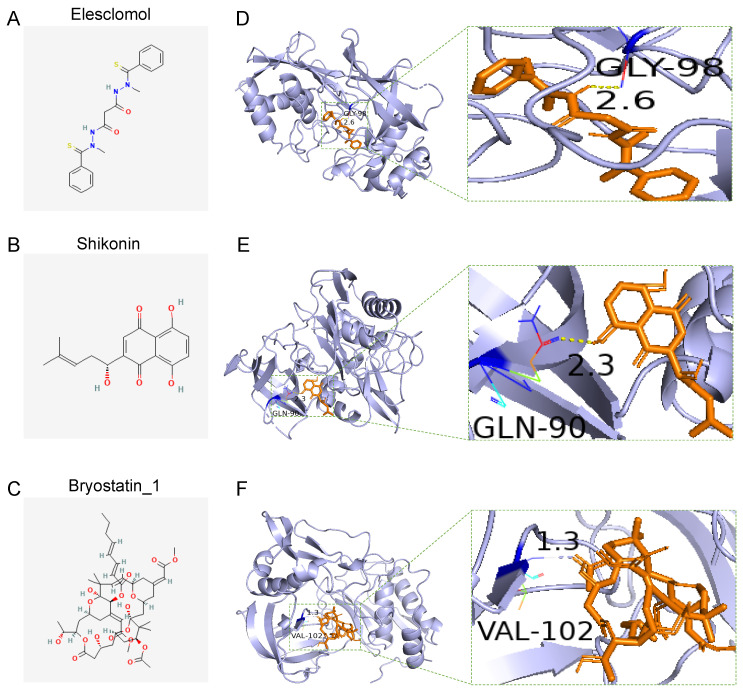
Molecular-docking analysis. (**A**–**C**) Elesclomol (**A**), Shikonin (**B**), and Bryostatin 1 (**C**) were screened-out by drug sensitivity analysis. Note that drug sensitivity analysis is shown in Supplementary Figure S3. (**D**–**F**) 3D structures and binding modes showing the formed hydrogen bonds between the predicted pocket of TIMP1 and Elesclomol (**D**), Shikonin (**E**), and Bryostatin_1 (**F**).

To further explore the difference in the drug-resistance potentials between the high- and low-risk groups, we compared the estimated IC50 levels of small molecule drugs in the two groups with the “pRRophetic” R package. Among these potential drugs, we found that the sensitivity of elesclomol, shikonin, and bryostatin-1 showed significant differences between groups (*p* < 0.001; Supplementary Figure S3). To further investigate if the potential drugs could bind to TIMP1, the structures of these drugs were downloaded from the PubChem database (Figure 11A–C), and then they were docked to TIMP1. The molecular-docking results showed relatively high affinity scores between the drugs and TIMP1 (Supplementary Table S4). The formation of hydrogen bonds could be observed between the drugs and the predicted pocket of TIMP1 (Figure 11D–F).

## 4. Discussion

Currently, researchers have developed considerable novel prognostic models for colon cancer based on clinical features, immune cell infiltration, subgroups of the TMN stage, the expression level of mRNA, and non-coding RNA [24,25,26,27]. However, due to the heterogeneity of the disease, prognostic models with high specificity and accuracy are still rare; therefore, more models need to be developed and tested in clinical practice.

MTRGs are a set of membrane tension-related genes that may be involved in carcinogenesis and metastasis. In this study, we retrieved 43 MTRGs from previously published literature. Then, we developed a novel 4-MTRG prognostic model for colon cancer based on differential gene analysis and Cox regression. The signature consists of four genes: EGFR, TIMP1, CAV1, and CDC42. EGFR is a known regulator of colon cancer contributing to tumor carcinogenesis and progression [28]. A recent study found that macrophages marked with phosphorylated EGFR play a crucial role in the development of the inflammation-mediated stages of colon carcinogenesis [29]. Research also revealed that EGFR cooperates with other genes such as HER-2 and YB-1 to promote cell growth and survival [30,31]. As an MTRG, EGFR may regulate membrane tension during its physiological trafficking. Therefore, cancerous cells could take advantage of such a functional link to enhance their oncogenic influence [17]. TIMP1 is a key element in the regulation of ECM remodeling and is secreted through the plasma membrane via exocytosis [32]. Meanwhile, the expression of TIMP1 and CAV1 increases in cirrhosis and hepatocellular carcinoma [12]. This may suggest that these MTRGs may cooperate in regulating the stiffness of tumors. CAV1 is considered a cell surface protein involved in the formation of caveolae, small invaginations of the plasma membrane [33]. An elevated level of CAV1 expression may contribute to colorectal tumor progression by enhancing aerobic glycolysis in colon cancer cells [34]. However, the regulatory mechanism of CAV1 in colon cancer in terms of membrane tension is not well understood. CDC42 has emerged as a key player in cancer metastasis due to its roles in regulating cell division and actin cytoskeletal rearrangements [35]. CDC42 accumulates at the cell’s leading edge dependent on membrane traffic, and controls the dilation of the exocytotic fusion pore by regulating membrane tension [36,37]. Though CDC42 was reported highly expressed in colon cancer and was downregulated by the potential tumor suppressor gene ID4, its role in the metastasis of colon cancer is still unknown [38].

Based on the risk score calculated in the prognostic model, we divided the patients in the TGCA cohort into two risk groups, considered high- and low-risk. The model exhibited accuracy and robustness in predicting survival rates in the TGCA cohort and two validation cohorts. Our 4-MTRG prognostic model is easy to use and might increase the accuracy of survival probability predictions for colon cancer patients. Then, we conducted a series of enrichment analyses and immune-related analyses to further evaluate the mechanism of how this signature regulates colon cancer and interacts with the microenvironment. The result of the GSEA analysis revealed that typical inflammatory pathways such as JAK-STAT3 and novel pathways such as PD-1 rank highly in the high-risk group. Essential inflammatory pathways such as STAT3 are understood to be drivers of inflammation and tumorigenesis, and diving into the crosstalk of these pathways advances our understanding of the complex links between inflammation and colon cancer [39]. In the immune-related analysis, first, we applied the CIBERSOFT algorithm to depict the immune cell infiltration landscape, and we observed less infiltration of CD4 T cells and more infiltration of M0 macrophages and regulatory T cells (Treg) in the patients of the high-risk group. CD4 T cells promote colon cancer stemness via the regulation of stemness genes, which negatively affects patient outcome [40]. The role of Tregs in the progression of colon cancer is controversial: it was shown that Tregs present antitumor immunity through the production of cytokines, such as TGF-β in the early stage, while the infiltration of tumors by Tregs confers growth and metastatic advantages by inhibiting antitumor immunity as the stage progresses [41]. The significance of the high infiltration of M0 macrophages is yet unknown. In the subsequent ssGSEA analysis, we noticed that immune cells in the high-risk group were more active, and many immune-related hallmarks were enriched, such as the T cell co-stimulation pathway and the type II IFN response pathway. This observation corresponds to a recent study showing that different T-cell subsets can express IFN-γ, thereby altering the immune responses in the colorectal cancer microenvironment [42]. Then, four immune-related scores were adopted to evaluate the immune status of the two groups, and the result showed that scores in the high-risk group were significantly high. Previous studies revealed that high immune and stromal scores as well as a high degree of infiltration in macrophages were associated with a poor prognosis of colon cancer [43]. In addition, we evaluated the relationship between the risk score and ICIs, and the relationship between the risk score and the cuproptosis-related genes. The result suggests that patients with high-risk scores might benefit most from drugs targeting ICIs and cuproptosis-related genes.

In the scRNA-seq analysis, we described the landscape of the samples and annotated the clusters with their typical cell markers. We noticed that all the 4-MTRGs exhibited expression in the epithelial mix. Therefore, we re-clustered the epithelial mix and conducted a pseudo-time analysis. The result showed that over half of the epithelial mix was estimated to be malignant by the “infercnv” package. In particular, TIMP1 was the only MTRG that responded to the shift of stage and had a relative threefold increase in expression with the progression of time. TIMP1 is not only considered a prognostic biomarker for various cancers [44] but promotes tumor progression. For example, an increased degree of TIMP1 expression promotes the in vivo growth of both cancer types and stimulates the accumulation of cancer-associated fibroblasts [45]. However, how membrane tension is involved in the dysfunction of TIMP1 is still unknown. Finally, we compared the drug sensitivity levels between the high- and low-risk groups and selected three potential drugs, elesclomol, shikonin, and bryostatin-1. Then, we conducted molecular docking on these drugs to TIMP1. The result showed that all three drugs had an adequate affinity score and the formation of hydrogen bonds was observed. TIMP1 was found to decrease tumor cell sensitivity to multiple anticancer drugs by activating downstream pathways and it also exhibited anti-apoptotic activity [46]. Therefore, discovering and developing novel drugs dependent on alternative pathways of cell death programming is essential. Copper metabolism has a vital role in tumorigenesis and elesclomol, as a copper chelator, could inhibit colon cancer cells by targeting ATP7A and regulating ferroptosis [47]. The second drug, shikonin, was reported to induce apoptosis and autophagy in colon cancer cells by targeting galectin-1 and the JNK-signaling pathway [48]; however, its effect on TIMP1 has not been tested. Overall, we hope these drugs will bind to TIMP1, relieving the progression of colon cancer and the occurrence of drug resistance.

There are still some limitations that must be addressed. Given that our results are based on sequencing datasets and computational simulations, further experimental and clinical research is needed to evaluate the effect of our 4-MTRG prognostic model. Moreover, the underlying mechanisms of these four selected genes in our model should be further explored to help us understand the intrinsic mechanisms involved in the tumorigenesis, progression, and metastasis of colon cancer. Further research on the dynamics of the plasma membrane and its relationship with broad-sense carcinogenesis and metastasis is also necessary.

## 5. Conclusions

This study demonstrates that MTRGs can be used to classify colon cancer patients based on different clinical and molecular features. A robust 4-MTRG signature model has been presented, which can predict the prognosis of colon cancer patients. Moreover, this study also found that TIMP1, as a membrane tension-related gene, may serve as a therapeutic target in colon cancer patients. The findings of our study provide insights in predicting the prognosis of colon cancer patients, and the drugs we have selected might contribute to treatments in clinical practice.

## Data Availability

The original contributions presented in the study are included in the Supplementary Material. Further inquiries can be directed to the corresponding authors.

## Supplementary Materials

The following supporting information can be downloaded at: https://www.mdpi.com/article/10.3390/vaccines10091562/s1, Table S1. Demographic information of COAD patients in the present study. Table S2. 44 membrane tension-related genes. Table S3. Multivariate COX regression analysis results of model genes. Table S4. Molecular docking scores of the three compounds. Figure S1. The boxplots for the comparison of the immune checkpoints genes between the high-risk and low-risk groups in the colon cancer patients. Figure S2. InferCNV analysis in epithelial cell subsets. Figure S3. Drug sensitivity analysis in the high-risk and low risk groups in colon cancer patients.
