# Supplementary material for: Integration of Bulk and Single-Cell RNA-Seq Data to Construct a Prognostic Model of Membrane Tension-Related Genes for Colon Cancer
Source: Vaccines (Basel). 2022 Sep 19;10(9):1562. doi: 10.3390/vaccines10091562 (PMC9506318; doi:10.3390/vaccines10091562)
Supplement: Supplementary file 1 [file vaccines-10-01562-s001.zip › vaccines-1900262-supplementary.pdf]

Supplementary Material

Supplementary Table S1 Demographic information of COAD patients in the present study.

Variables	TCGA-COAD (Training)	GSE14333 (Validation1)	GSE103479 (Validation2）
**Total**	426	223	154
**Gender**			
Female	198	105	68
Male	228	118	86
**Age**			
<60	120	60	28
≥60	306	163	124
**AJCC stage**			
I	73	-	0
II	165	-	82
III	119	-	72
VI	58	-	0
Unknow	11	-	0
**T stage**			
T1	10	-	1
T2	74	-	6
T3	291	-	109
T4	50	-	38
Tis	1	-	0
**M stage**			
M0	317	-	85
M1	58	-	0
Mx	44	-	69
Unknow	7	-	0
**N stage**			
N0	253	-	82

N1	99	-	50
N2	74	-	22

Supplementary Table S2 44 membrane tension-related genes.

Number	Gene	Number	Gene
1	RHOA	23	FNBP1
2	CTNNBIP1	24	MTSS1L
3	EZR	25	TRIP10
4	RDX	26	CRTC2
5	MSN	27	CFL1
6	MYO1A	28	CAPZB
7	GSK3b	29	DIAPH1
8	FGFR1	30	MYH9
9	GBF1	31	MYH10
10	BAIAP2	32	PIEZO1
11	ARF1	33	SDC4
12	CDC42	34	PECAM1
13	ACTR2	35	CDH1
14	ACTR3	36	ITGB1
15	PICK1	37	FERMT2
16	EGF	38	TIMP1
17	CHMP4B	39	CAV1
18	EGFR	40	DNM2
19	TSG101	41	MYO1B
20	ALIX	42	ARHGAP26
21	SLK	43	FLNA
22	STK10	44	MYO1C

Supplementary Table S3 Multivariate COX regression analysis results of model genes.

**Gene**	**Coef**	**HR**	**HR.95L**	**HR.95H**	***p* Value**
CDC42	-0.04318	0.957736	0.916508	1.000819	0.054418
EGFR	0.032753	1.033295	1.004365	1.063058	0.023785
TIMP1	0.001374	1.001375	0.999951	1.002802	0.058403
CAV1	0.011181	1.011243	1.001203	1.021385	0.028088

Supplementary Table S4 Molecular docking scores of the three compounds.

Rank	PubChem CID	Compound name	Affinity score (kcal/mol)
1	300471	Elesclomol	-7.787
2	479503	Shikonin	-7.2
3	5280757	Bryostatin_1	-7.1

Supplementary Figures

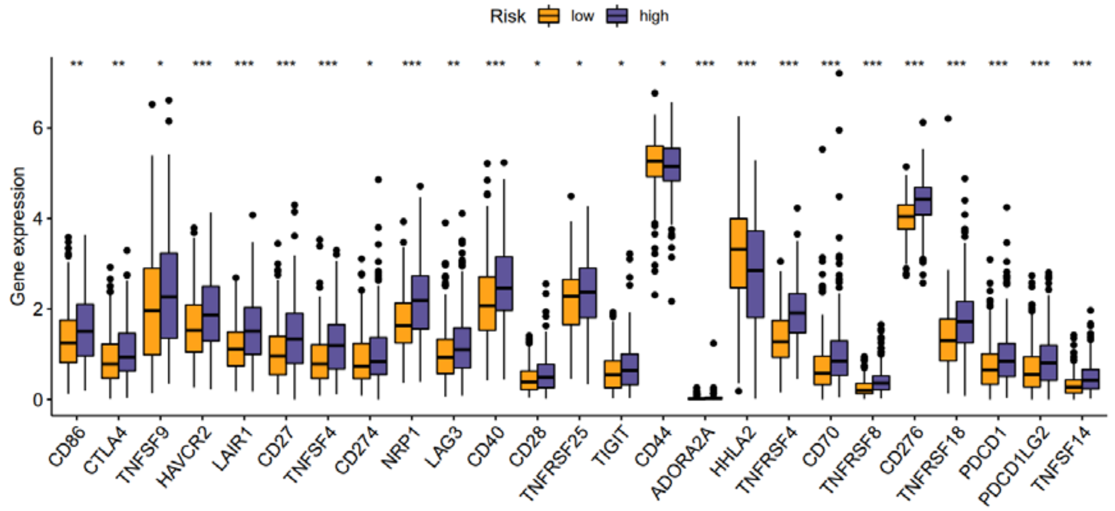

Supplementary Figure S1 The boxplots for the comparison of the immune checkpoints genes between the high-risk and low-risk groups in the colon cancer patients. *p < 0.05, **p < 0.01, and ***p < 0.001.

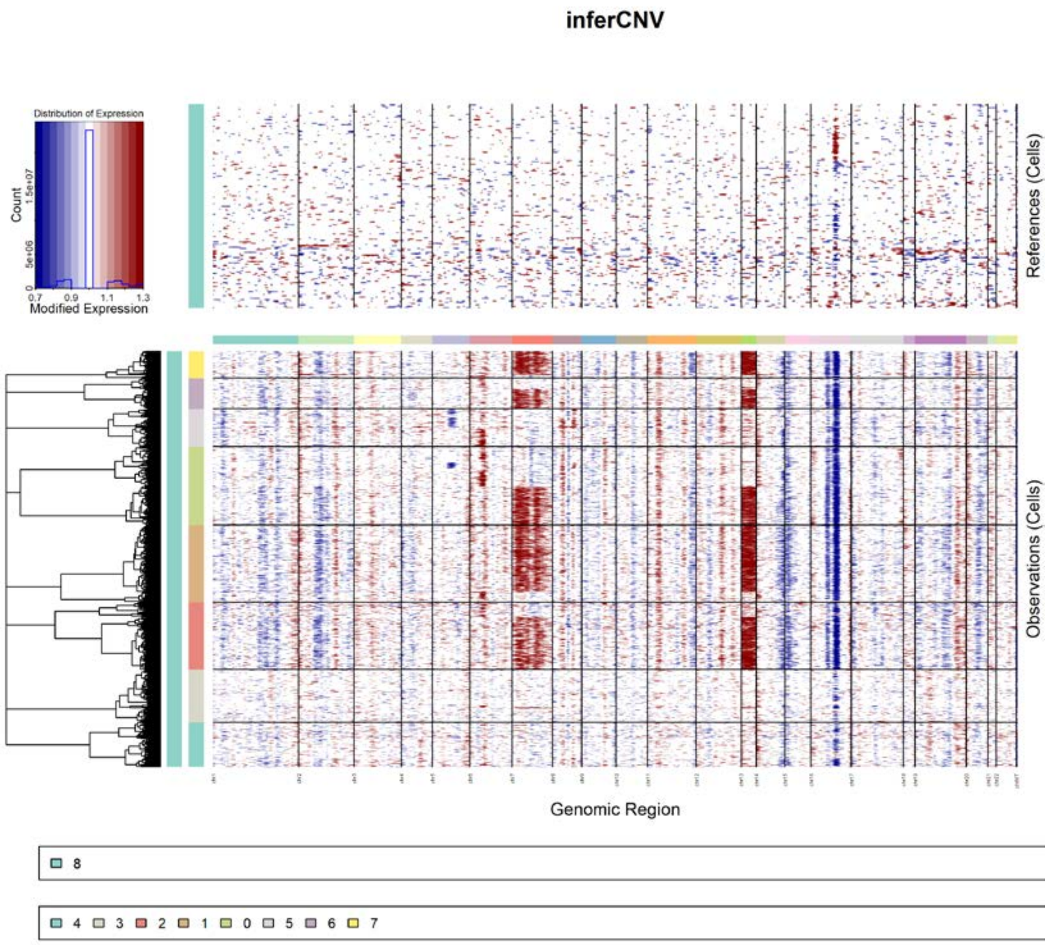

Supplementary Figure S2 InferCNV analysis in epithelial cell subsets.

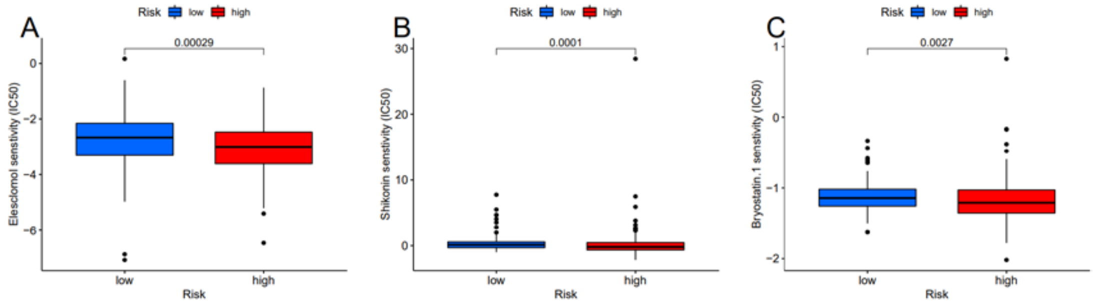

Supplementary Figure S3 Drug sensitivity analysis in the high-risk and low risk groups in colon cancer patients. (A) Elesclomol (B), Shikonin (C) and Bryostatin 1.
